# SarS Is a Repressor of Staphylococcus aureus Bicomponent Pore-Forming Leukocidins

**DOI:** 10.1128/iai.00532-22

**Published:** 2023-03-20

**Authors:** Exene E. Anderson, Sophie Dyzenhaus, Juliana K. Ilmain, Mitchell J. Sullivan, Harm van Bakel, Victor J. Torres

**Affiliations:** a Department of Microbiology, New York University Grossman School of Medicine, New York, New York, USA; b Department of Genetics and Genomic Sciences, Icahn School of Medicine at Mount Sinai, New York, New York, USA; c Icahn Genomics Institute, Icahn School of Medicine at Mount Sinai, New York, New York, USA; University of Illinois at Chicago

**Keywords:** MRSA, cytotoxins, leukocidins, pathogenesis, regulation, toxin

## Abstract

Staphylococcus aureus is a successful pathogen that produces a wide range of virulence factors that it uses to subvert and suppress the immune system. These include the bicomponent pore-forming leukocidins. How the expression of these toxins is regulated is not completely understood. Here, we describe a screen to identify transcription factors involved in the regulation of leukocidins. The most prominent discovery from this screen is that SarS, a known transcription factor which had previously been described as a repressor of alpha-toxin expression, was found to be a potent repressor of leukocidins LukED and LukSF-PV. We found that inactivating *sarS* resulted in increased virulence both in an *ex vivo* model using primary human neutrophils and in an *in vivo* infection model in mice. Further experimentation revealed that SarS represses leukocidins by serving as an activator of Rot, a critical repressor of toxins, as well as by directly binding and repressing the leukocidin promoters. By studying contemporary clinical isolates, we identified naturally occurring mutations in the *sarS* promoter that resulted in overexpression of *sarS* and increased repression of leukocidins in USA300 bloodstream clinical isolates. Overall, these data establish SarS as an important repressor of leukocidins and expand our understanding of how these virulence factors are being regulated *in vitro* and *in vivo* by S. aureus.

## INTRODUCTION

Staphylococcus aureus is an opportunistic pathogen that poses a serious threat to human health worldwide, resulting in both hospital- and community-associated infections. Around one in three healthy individuals are colonized with S. aureus on the skin and in the nares (1), where it is often a harmless commensal. However, when S. aureus gains access to the bloodstream and deeper tissues, it can cause a wide range of serious diseases, including pneumonia, infective endocarditis, bacteremia, osteomyelitis, and skin and soft tissue infections (2). One reason S. aureus is such a successful pathogen is that it possesses a wide range of virulence factors (3) which it deploys to disrupt and weaken the host’s immune system (4, 5). One subset of these virulence factors are the bicomponent leukocidins, which include LukAB (also known as LukHG), LukED, LukSF-PV (also known as PVL), HlgAB, and HlgCB (6, 7). The leukocidins are of particular importance for pathogenesis, as they provide protection from immune-mediated killing and modulate the host immune responses (8). Leukocidins primarily target leukocytes but can also bind and lyse erythrocytes and endothelial cells (9).

Toxin production by S. aureus is a highly regulated process that responds to quorum via the accessory gene regulatory (Agr) system (10). This two-component quorum-sensing system is a master regulator of virulence and increases production of exotoxins and other secreted proteins, while decreasing the expression of cell wall-associated proteins. As bacterial density increases and reaches quorum, the accumulation of an autoinducing peptide results in the activation of the Agr system, which leads to the production of more autoinducing peptides in a positive-feedback loop (10, 11). The effector molecule of the Agr system, RNAIII, is also transcribed. RNAIII is a regulatory RNA that targets downstream genes (12, 13). One critical target that RNAIII represses is the repressor of toxins (Rot) (14). Rot directly represses toxin production in S. aureus by binding to the promoter regions of S. aureus toxin genes and inhibiting their expression (15–17). Rot has also been shown to indirectly repress toxin production by inhibiting *saeRS* expression (16, 18). The SaeRS two-component system is a critical activator of leukocidin expression (19–25) and responds to environmental cues and molecules encountered *in vivo* (21, 26, 27).

The ability to regulate virulence factors with a high level of specificity and in response to the different environments that the bacterium is occupying is thought to be key for S. aureus pathogenesis (28, 29). The regulatory networks that control the expression of virulence factors in S. aureus is intricate and interwoven, and we still do not fully understand the complexities of the entire system. Further characterization of how virulence factors are regulated will result in a better understanding of S. aureus pathogenesis.

In this study, we aimed to identify and characterize transcriptional activators and repressors of leukocidins. We identified SarS as a potent repressor of leukocidin activity and demonstrated that the inactivation of *sarS* led to an increase in toxin expression, production, and cytotoxicity of S. aureus against primary human neutrophils. We determined two different pathways through which SarS represses leukocidins: direct repression by binding to the target promoters and indirect repression by acting on Rot production. Moreover, we found mutations in clinical isolates that alter the expression of *sarS*, impacting the virulence potential of S. aureus. Overall, these data implicate SarS as a key member of the ever-growing network of transcriptional activators and repressors that contribute to the regulation of S. aureus leukocidins.

## RESULTS

### Screen to identify transcriptional regulators that alter S. aureus leukocidin promoter activity.

**FIG 1:**
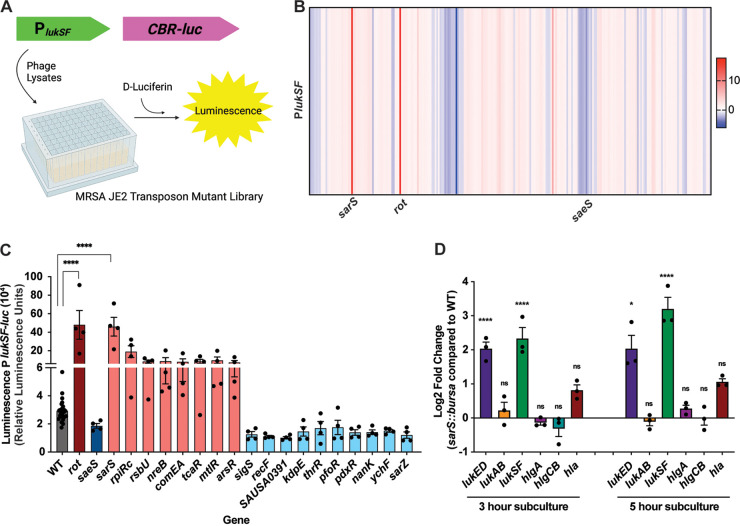
Screen to identify transcriptional regulators that alter S. aureus leukocidin promoter activity. (A) The JE2 regulatory sublibrary was phage transduced to have the *lukSF-PV* promoter driving the expression of *CBR-luc*. (B) Heat map of the fold change P*lukSF-PV* luminescence compared to the wild-type JE2 strain in stationary-phase cultures, representing the average of four independent experiments. (C) Top changes in *lukSF-PV* promoter activity compared to the wild type, measured by luminescence. Statistical analysis was performed using 2-way ANOVA with multiple comparisons. Error bars show standard errors of the mean. (D) Log_2_ fold change of pore-forming toxin transcript levels measured by qRT-PCR in the *sarS*::*bursa* strain compared to wild-type AH-LAC in exponential and stationary phases of growth (*n* = 3). Statistical analysis was performed using 2-way ANOVA with multiple comparisons. Error bars show standard errors of the mean. *, *P* < 0.05; **, *P* < 0.01; ***, *P* < 0.001; ****, *P* < 0.0001. ns, not significant.

To identify transcriptional factors that regulate S. aureus leukocidins, we conducted a screen to measure *lukSF-PV* promoter activity in transposon mutants from the Nebraska transposon library (30), which was constructed in S. aureus strain JE2. JE2 is in the USA300 background, which is the most prevalent lineage in the current epidemic of community-associated methicillin-resistant S. aureus (MRSA) (31). To focus our search on genes that could be directly involved in virulence regulation, we utilized a previously described regulator sublibrary (32), which includes 250 mutants in genes likely to be involved in transcription and translation. We transduced these mutants with a plasmid containing a *lukSF-PV* promoter fusion driving the expression of the click beetle red luciferase (*CBR*-*luc*) (33). *lukSF-PV* was selected for this screen due to its high levels of expression *in vitro* (32), which would allow us to more easily observe change in expression between the transposon mutants and the wild-type (WT) strain. Following overnight growth, the reporter library was subcultured for 5 h in deep-well 96-well plates to stationary phase. We then added d-luciferin and measured luminescence as a readout for promoter activity (Fig. 1A).

Compared to the wild-type JE2 strain, we observed increased expression of the *lukSF-PV* reporter in the *rot* mutant (*rot*::*bursa*) and decreased expression in the *saeS* mutant (*saeS*::*bursa*), validating the screen. Altogether, the screen identified 27 mutants that had at least a 2-fold increase in promoter activity compared to the wild-type strain and 10 mutants that had at least 2-fold less promoter activity than wild-type JE2 (Fig. 1B; see also Table S1A in the supplemental material). We next determined which mutants had the largest differences in promoter activity and focused on genes that had features that would suggest that they were involved in the direct repression or activation of the toxin, such as DNA-binding motifs. This analysis resulted in 8 putative repressors and 10 putative activators of *lukSF-PV* promoter activity (Fig. 1C). Of note, all these mutants had upregulation or downregulation of promoter activity similar to that of either the *rot*::*bursa* strain or the *saeS*::*bursa* strain, respectively.

The mutant strain that had the highest level of *lukSF-PV* promoter activity was the *sarS*::*bursa* strain. SarS (also known as SarH1) is a member of the SarA family of S. aureus regulators (34, 35). The regulation of *sarS* is highly complex: *sarS* is repressed by SarA, MgrA, and Agr (34–36), while it is activated by SarT (37), Rot (38), and TcaR (39) and posttranscriptionally activated by *gdpS* (40). SarS is an activator of *spa* (encodes protein A), which is located directly downstream of the *sarS* locus (34). This activation is part of a regulatory cascade involving Agr-mediated regulation of SarT, which, in turn, activates SarS to upregulate *spa* expression (25). SarS is also an activator of the protease ScpA (41). Additionally, SarS has been shown to be a repressor of *hla*, the gene encoding alpha-toxin (34), as well as other virulence factors, including the protease-encoding genes *aur*, *ssp*, and *spl* (34, 41, 42), and the exfoliative toxin-encoding gene *eta* (43). Lastly, SarS has been shown to bind directly to the promoter regions of *hla*, *spa*, and *ssp* (34).

To further validate the high levels of *lukSF-PV* promoter activity that we observed in the *sarS*::*bursa* strain, we transduced the transposon into the USA300 AH-LAC background (44) and measured toxin transcript levels of all five leukocidins and alpha-toxin via reverse transcription-quantitative PCR (qRT-PCR), at both the exponential and stationary phases (Fig. 1D). We observed a significant increase in transcript levels of *lukED* and *lukSF-PV* in the *sarS*::*bursa* strain compared to the wild type at both time points, further establishing SarS as a repressor of leukocidins. Of note, we observed only a modest increase in *hla* transcript in *sarS*::*bursa* at both time points.

### SarS represses *lukED* and *lukSF-PV* expression and production.

**FIG 2:**
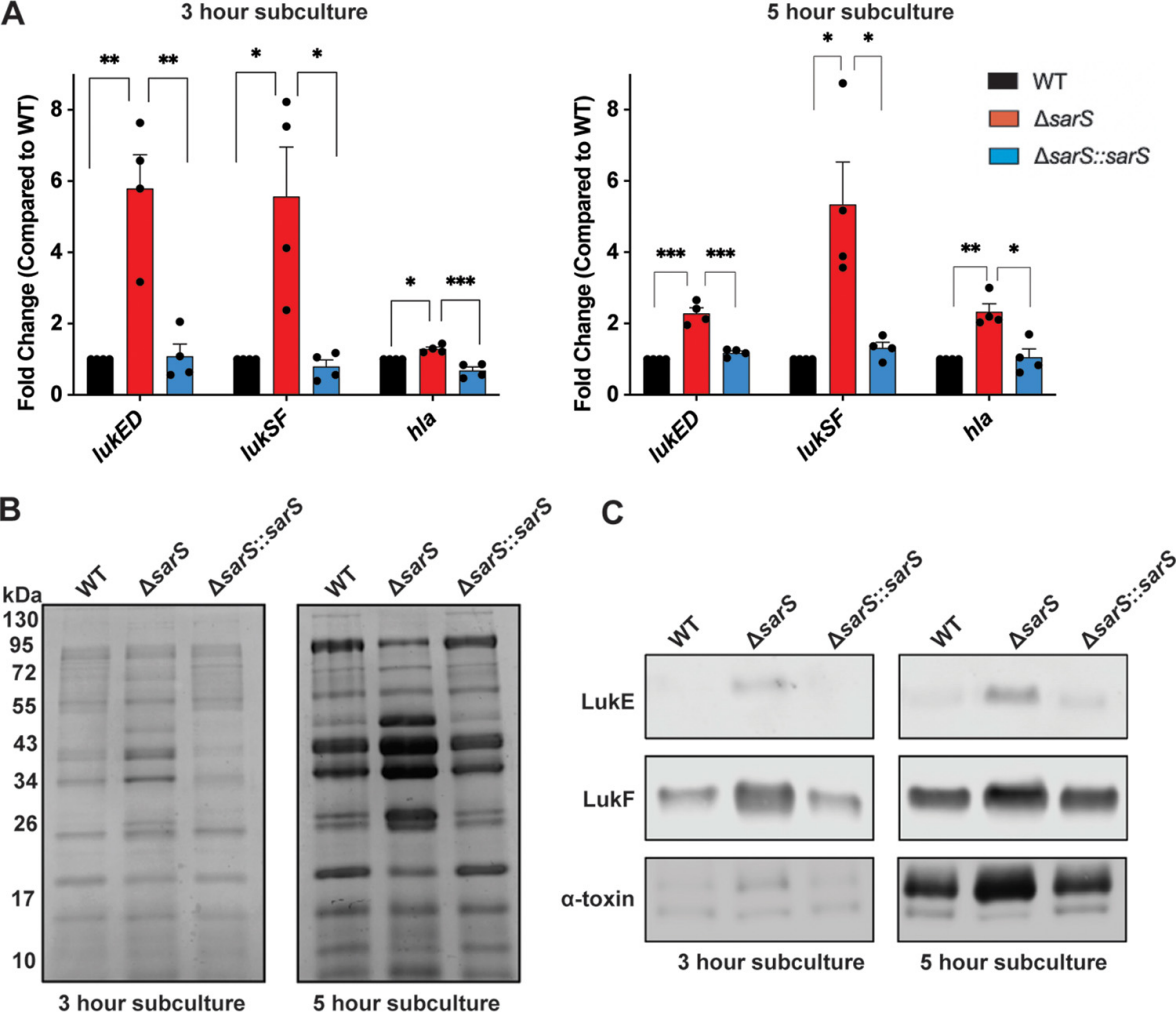
SarS represses *lukED* and *lukSF-PV* expression and production. (A) Toxin transcript levels measured by qRT-PCR show higher levels of leukocidin expression at both exponential and stationary phases in the Δ*sarS* strain compared to wild-type AH-LAC or the Δ*sarS*::*sarS* strain. Statistical analysis was performed using 2-way ANOVA with multiple comparisons. Error bars show standard errors of the mean (*n* = 4). (B) Supernatants collected from OD-normalized wild-type AH-LAC and Δ*sarS* and Δ*sarS*::*sarS* strains in exponential and stationary phases show altered exoprotein profiles (representative Coomassie-stained gels are shown). (C) Immunoblots of samples from panel B demonstrate that the Δ*sarS* strain produces greater amounts of LukE, LukF-PV, and alpha-toxin at both exponential and stationary phases of growth. *, *P* < 0.05; **, *P* < 0.01; ***, *P* < 0.001.

To further investigate the role of SarS in toxin regulation, we generated a pair of isogenic *sarS* deletion and complementation strains. The Δ*sarS* strain was made by phage transducing the *sarS*::*erm* mutation, described by Cheung et al. (35), into AH-LAC, while the Δ*sarS*::*sarS* complementation strain was created using pIMAY* (45), in which *sarS*::*erm* was replaced with wild-type *sarS*. As with the transposon strain, we observed a significant increase in *lukED* and *lukSF-PV* transcripts in the *sarS* deletion strain compared to the wild-type strain (Fig. 2A). Again, we detected a modest increase in *hla* transcripts as with the transposon strain. Importantly, the increased toxin levels were repressed to wild-type levels in the complementation strain. We observed that *lukED* and *lukSF-PV* transcripts were more highly upregulated in the Δ*sarS* strain than the *hla* transcript, suggesting that SarS represses these toxins to a greater extent than it represses *hla*.

We next investigated the impact of SarS on toxin production. We observed altered exoprotein profiles when *sarS* was deleted and noticed increased abundances of proteins that corresponded to the sizes of the two components of the leukocidins (~35 kDa and ~43 kDa) (Fig. 2B). Of note, these changes were restored to wild-type levels in the complementation strain. To directly evaluate the impact of deleting *sarS* on the production of leukocidins, we performed Western blotting using toxin-specific antibodies. We observed higher levels of LukE, LukF-PV, and alpha-toxin at both 3 and 5 h in the Δ*sarS* strain, a phenotype that was complemented by restoring *sarS* (Fig. 2C). Altogether, these data demonstrate that SarS represses *lukED* and *lukSF-PV* expression, leading to decreased toxin production.

### SarS represses toxins dually through activation of Rot and direct binding of toxin promoters.

**FIG 3:**
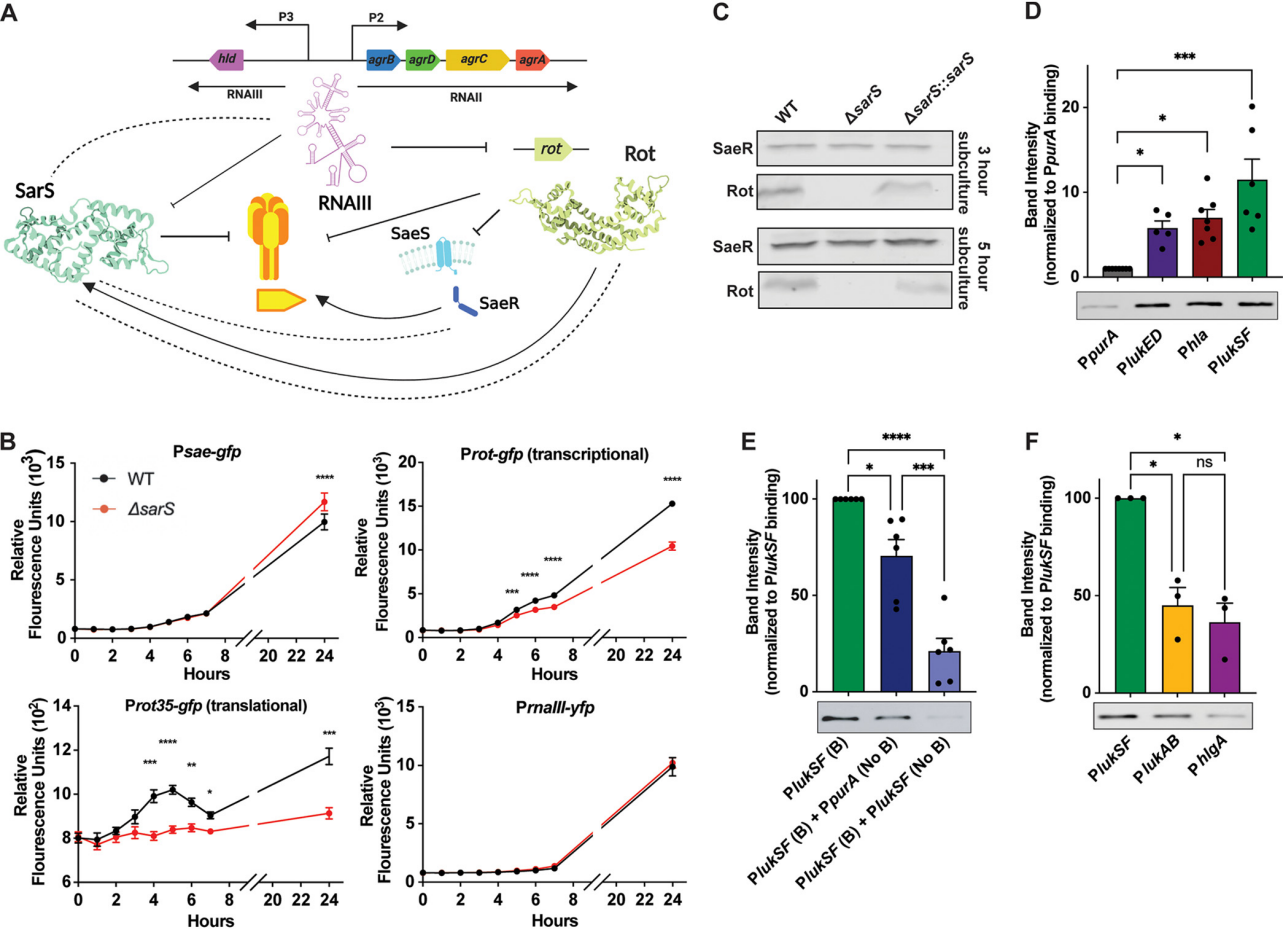
SarS represses toxins dually through the activation of Rot and the direct binding of toxin promoters. (A) Schematic showing the roles of RNAIII, Rot, and SaeRS in the regulation of leukocidins as well as *sarS*. Dotted lines indicate potential interactions (repression or activation) that may exist but are yet to be shown and will be tested between the regulators and SarS. (B) Time course comparing the expression of *sae*, *rot*, *rot* plus 35 amino acids, and *rnaIII* promoters fused to either GFP or yellow fluorescent protein (YFP). The values are averages of two independent experiments, with a total of 11 independent colonies of each strain. Statistical analysis was performed using 2-way ANOVA with multiple comparisons. Error bars show standard errors of the mean. (C) Western blots using the wild-type AH-LAC and Δ*sarS* and Δ*sarS*::*sarS* strains during exponential and stationary growth for Rot and SaeR (representative blots are shown). (D) Purified His-tagged SarS binds to the biotinylated *lukED*, *hla*, and *lukSF-PV* promoters more strongly than to the biotinylated negative-control *purA* promoter (800 fmol). Band intensity was normalized to P*purA* by converting P*purA* band intensity to a value of 1 across all experiments and calculating the values of the other bands relative to the P*purA* value. (E) SarS binding to P*lukSF-PV* [P*lukSF* (B)] can be competed off with nonbiotinylated P*lukSF-PV* DNA [P*lukSF* (No B)] to a greater extent than nonbiotinylated P*purA* DNA [P*purA* (No B)] when added at the same concentration as the biotinylated *lukSF-PV* promoter DNA (*n* = 6). Band intensity was normalized to P*lukSF-PV* by converting P*lukSF-PV* band intensity to a value of 100 across all experiments and calculating the values of the other bands relative to the P*lukSF-PV* value. (F) SarS binds significantly better to the *lukSF-PV* promoter than to the *lukAB* or *hlgA* promoter. Statistical analysis was performed using 2-way ANOVA with multiple comparisons. Error bars show standard errors of the mean. *, *P* < 0.05; **, *P* < 0.01; ***, *P* < 0.001; ****, *P* < 0.0001.

To determine how SarS represses the leukocidins, we first investigated if SarS was regulating any of the known regulators of toxin expression in S. aureus. This includes RNAIII, the effector molecule of the Agr system, the repressor of toxins Rot, and the activator of leukocidins, the SaeRS system (Fig. 3A). We measured the promoter activities using green fluorescent protein (GFP) fusions of these regulators in the wild type and *sarS* deletion strains. Additionally, a *rot* translational fusion was tested in which the promoter and the first 35 amino acids of the coding sequence were fused to GFP. We observed a repression of both the *rot* transcription and *rot* translation fusions in the Δ*sarS* strain, demonstrating that SarS is an activator of *rot* expression (Fig. 3B). In contrast, *sae* promoter activity was only minimally increased when *sarS* was deleted in late stationary phase. We observed no detectable change in the activation of the *rnaIII* promoter in the *sarS* mutant background.

To further investigate the impact of SarS on Rot and SaeR production, we performed Western blotting. We observed that in the absence of *sarS*, there was a notable decrease in the amount of Rot that S. aureus was producing at both the exponential and stationary phases (Fig. 3C). Complementation studies rescued Rot production, albeit to a slightly lower level than with the wild-type strain. These findings agree with the observed repression of the *rot* transcription and translation fusions in the Δ*sarS* strain. We saw no notable difference in the amount of SaeR in the wild-type, Δ*sarS*, or Δ*sarS*::*sarS* strain, which corroborated the GFP reporter data.

Having found that SarS can indirectly repress leukocidins through the activation of Rot, we wanted to determine if SarS could also directly repress toxins through the binding of their promoter regions. We performed DNA-binding experiments between the promoters of *lukED*, *lukSF-PV*, and *hla* and purified SarS. Biotinylated DNA probes were conjugated to streptavidin magnetic beads, and following incubation with His-tagged SarS, binding was detected via immunoblotting. We included the promoter for *purA* as a control to which SarS was not expected to bind based on the literature (34, 35). We found that SarS bound more strongly to the *lukED*, *lukSF-PV*, and *hla* promoters than to the negative-control *purA* promoter (Fig. 3D). Moreover, while nonbiotinylated *purA* DNA was able to only modestly outcompete binding of SarS to the *lukSF-PV* promoter, the SarS binding was competed off with nonbiotinylated *lukSF-PV* promoter DNA (Fig. 3E). Notably, SarS was able to bind to the *lukSF-PV* promoter significantly better than the *lukAB* or *hlgA* promoters (Fig. 3F), supporting the idea that SarS differentially regulates the leukocidins. Altogether, these results suggest that SarS can directly interact with the promoters of leukocidins, which may contribute to their repression.

### Deletion of *sarS* enhances S. aureus virulence.

**FIG 4:**
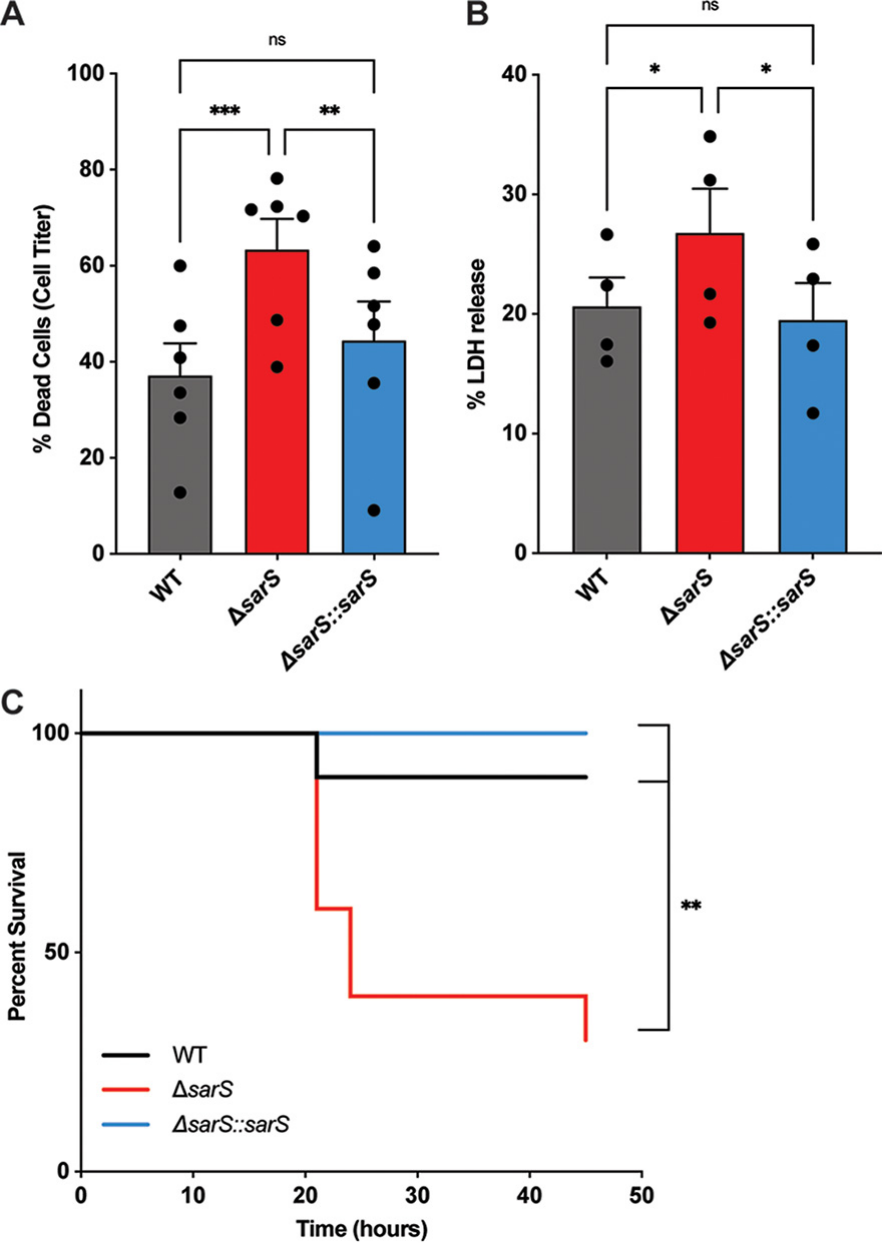
Deletion of *sarS* enhances S. aureus virulence. (A) Culture supernatants collected from the *sarS* deletion strain (1.25%) are more cytotoxic toward primary human PMNs than supernatants collected from the wild-type AH-LAC strain or the complemented Δ*sarS*::*sarS* strain. Cell death of the PMNs was measured with CellTiter. Each data point represents an individual donor (*n* = 6). Statistical analysis was performed using 2-way ANOVA with multiple comparisons. Error bars show standard errors of the mean. (B) Primary human PMNs infected with wild-type AH-LAC and Δ*sarS* and Δ*sarS*::*sarS* strains at an MOI of 100 are more susceptible to the Δ*sarS* strain than the wild-type or complemented strain. Cell death of the PMNs was measured with LDH release. Each data point represents an individual donor (*n* = 4). Statistical analysis was performed using 2-way ANOVA with multiple comparisons. Error bars show standard errors of the mean. (C) Eight-week-old female B6 mice were infected intraperitoneally with wild-type AH-LAC or Δ*sarS* or Δ*sarS*::*sarS* S. aureus at a concentration of 1 × 10^8^ CFU/mouse and monitored for survival (*n* = 10 mice per group). Statistical analysis was performed using log rank Mantel-Cox test. *, *P* < 0.05; **, *P* < 0.01; ***, *P* < 0.001.

Next we wanted to determine the role of SarS in the virulence potential of USA300. We measured the cytotoxicity of supernatants from the wild-type, *sarS* deletion, and complementation strains on primary human neutrophils. When *sarS* was deleted, there was significantly more killing of the neutrophils than with the wild-type strain, which we attributed to there being more toxins present in the supernatant (Fig. 4A). This phenotype was fully complemented in the Δ*sarS*::*sarS* strain. We also infected neutrophils with live wild-type, Δ*sarS*, and Δ*sarS*::*sarS* strains and measured neutrophil killing by the bacteria. Similar to the intoxication data from supernatants, we observed a significant increase in neutrophil death when the cells were infected with the Δ*sarS* strain compared to the wild-type or complementation strain (Fig. 4B).

To further understand the role of SarS in pathogenesis, an *in vivo* model of murine intraperitoneal infection was utilized. Mice were infected with the wild-type, Δ*sarS*, and Δ*sarS*::*sarS* strains and monitored for survival. Significantly more mice infected with the Δ*sarS* strain succumbed to the infection than the mice infected with the wild-type strain, a phenotype that was complemented by introducing a wild-type copy of *sarS* (Δ*sarS*::*sarS*) (Fig. 4C). Altogether, these data demonstrate that *sarS* is critical for USA300 pathogenesis.

### Natural mutations in the *sarS* promoter result in altered *sarS* expression and cytotoxicity.

**FIG 5:**
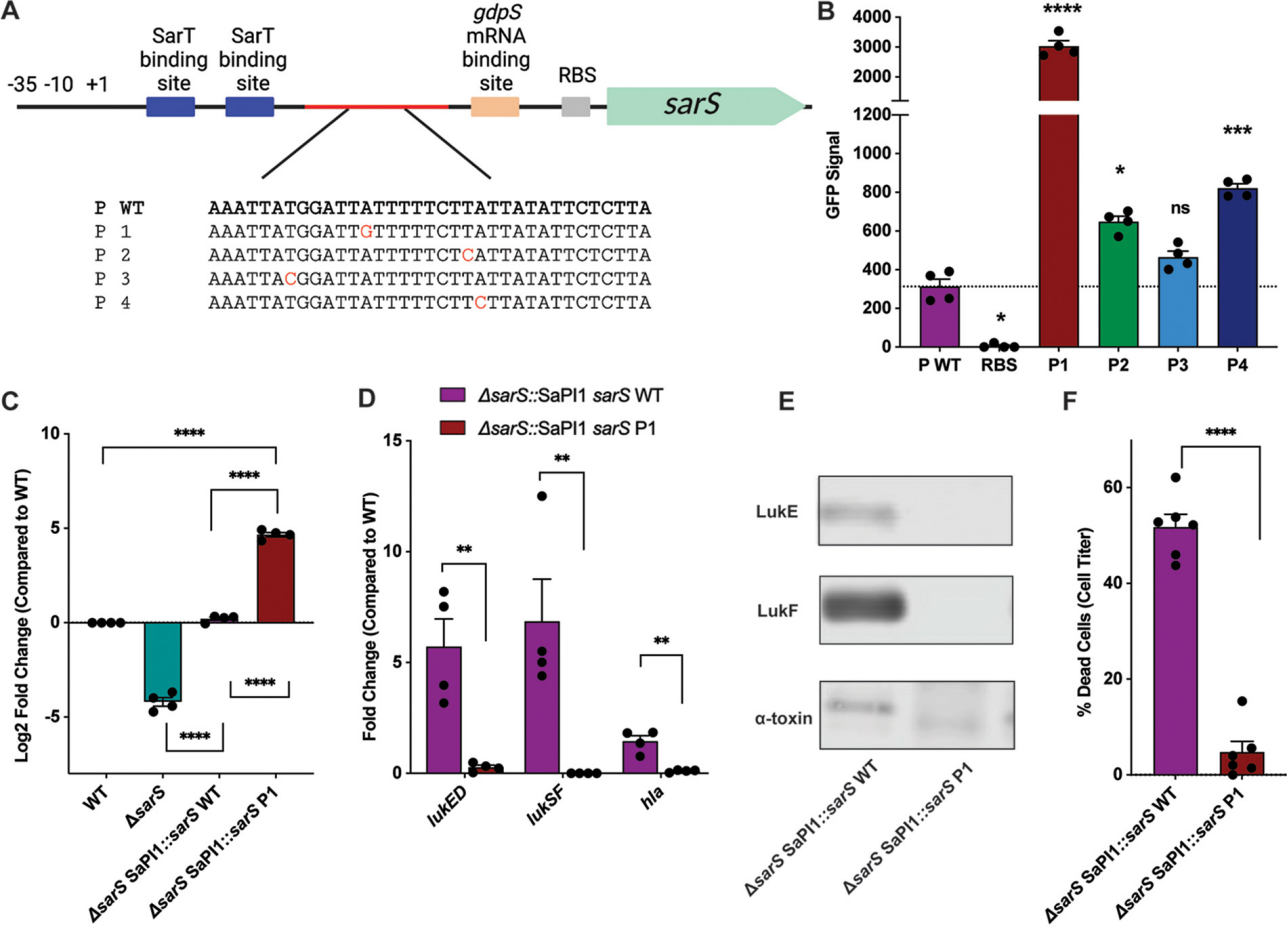
Bloodstream USA300 isolates harbor mutations in the *sarS* promoter. (A) USA300 bloodstream clinical isolates were found to have mutations in a 16-bp region of the *sarS* promoter. Shown is a schematic of *sarS* and upstream region with previously described features (37). “P WT” refers to the sequence found in AH-LAC. P1 to P4 are sequences found in clinical isolates. Mutated bases are highlighted in red. (B) Reporter strains in which GFP production was driven by the wild-type AH-LAC promoter or the mutant promoters (*n* = 4). (C) The wild-type and P1 promoters were cloned into the SaPI1 site in the Δ*sarS*::*sarS* strain and *sarS* transcript level was measured by qRT-PCR at exponential phase (*n* = 4). (D) Transcript levels of *lukED*, *lukSF-PV*, and *hla* were measured in the strains by qRT-PCR at exponential phase (*n* = 4). (E) Immunoblotting was performed for LukE, LukF-PV, and alpha-toxin. (F) Supernatants (2.5%) from the Δ*sarS* SapI1::*sarS* P1 strains were more cytotoxic than supernatants from the Δ*sarS* SapI1::*sarS* wild-type strain, as measured by CellTiter. Each data point represents an individual donor (*n* = 6). For all bar graphs, statistical analysis was performed using 2-way ANOVA with multiple comparisons. Error bars show standard errors of the mean. *, *P* < 0.05; **, *P* < 0.01; ***, *P* < 0.001; ****, *P* < 0.0001.

The identification of SarS as a potent repressor of leukocidins prompted us to examine if clinical isolates differ in their *sarS* sequences, which could impact S. aureus pathogenesis. We analyzed the genomes of a series of contemporary USA300 bloodstream isolates whose cytotoxicity against human neutrophils had previously been characterized (46). In doing this, we discovered several low-cytotoxicity isolates that harbored mutations in a 16-bp region within the *sarS* promoter (Fig. 5A). The mutations localized downstream of the putative translational start site and the SarT binding sites and upstream of the *gdpS* mRNA binding site. This encompasses a stretch of DNA that contains both the promoter and the 5′ untranslated region (UTR) of *sarS*. We hypothesized that these mutations resulted in an overexpression of *sarS*, which contributed to the observed decrease in cytotoxicity. To determine if this was the case, GFP reporter strains were constructed containing either these mutant promoters/5′ UTRs (P1 to P4), the USA300 reference strain promoter/5′ UTR (WT), or just the native *sarS* ribosomal binding sequence (RBS). We observed that several of the promoter/5′ UTR mutations led to a greater GFP signal, suggesting that indeed these mutations resulted in higher expression of *sarS* than the wild-type nucleotide sequence (Fig. 5B).

Next, we constructed strains in which Δ*sarS* was complemented with *sarS* driven by either the wild-type or the P1 promoter, both located at the pathogenicity island SaPI1 (47). The P1 promoter was chosen because it showed the largest increase in GFP signal compared to the wild-type promoter in the reporter assay. To test that the P1 promoter resulted in increased *sarS* expression, qRT-PCR was performed. We observed that the P1 mutation resulted in a significant increase in *sarS* mRNA compared to the wild-type and wild-type complemented strains (Fig. 5C).

We also examined toxin expression by qRT-PCR and observed a decrease in *lukED*, *lukSF-PV*, and *hla* transcript levels in the Δ*sarS* SaPI1::*sarS* P1 strain compared to the Δ*sarS* SaPI1::*sarS* WT strain (Fig. 5D). Consistent with the expression levels, Western blots for LukE, LukF-PV, and alpha-toxin revealed less production of all three toxins in the Δ*sarS* SaPI1::*sarS* P1 compared to the Δ*sarS* SaPI1::*sarS* WT strain (Fig. 5E).

We next examined how the *sarS* P1 promoter mutation impacted S. aureus cytotoxicity toward immune cells. We collected supernatants from the complemented strains and intoxicated primary human neutrophils. Supernatants from the Δ*sarS* SaPI1::*sarS* P1 strain were significantly less cytotoxic than supernatants from the Δ*sarS* SaPI1::*sarS* WT strain (Fig. 5F), demonstrating that the decrease in leukocidin production due to the *sarS* promoter mutations that occur in the bloodstream resulted in altered virulence patterns.

### USA300 isolates harbor mutations in the *sarS* promoter.

**FIG 6:**
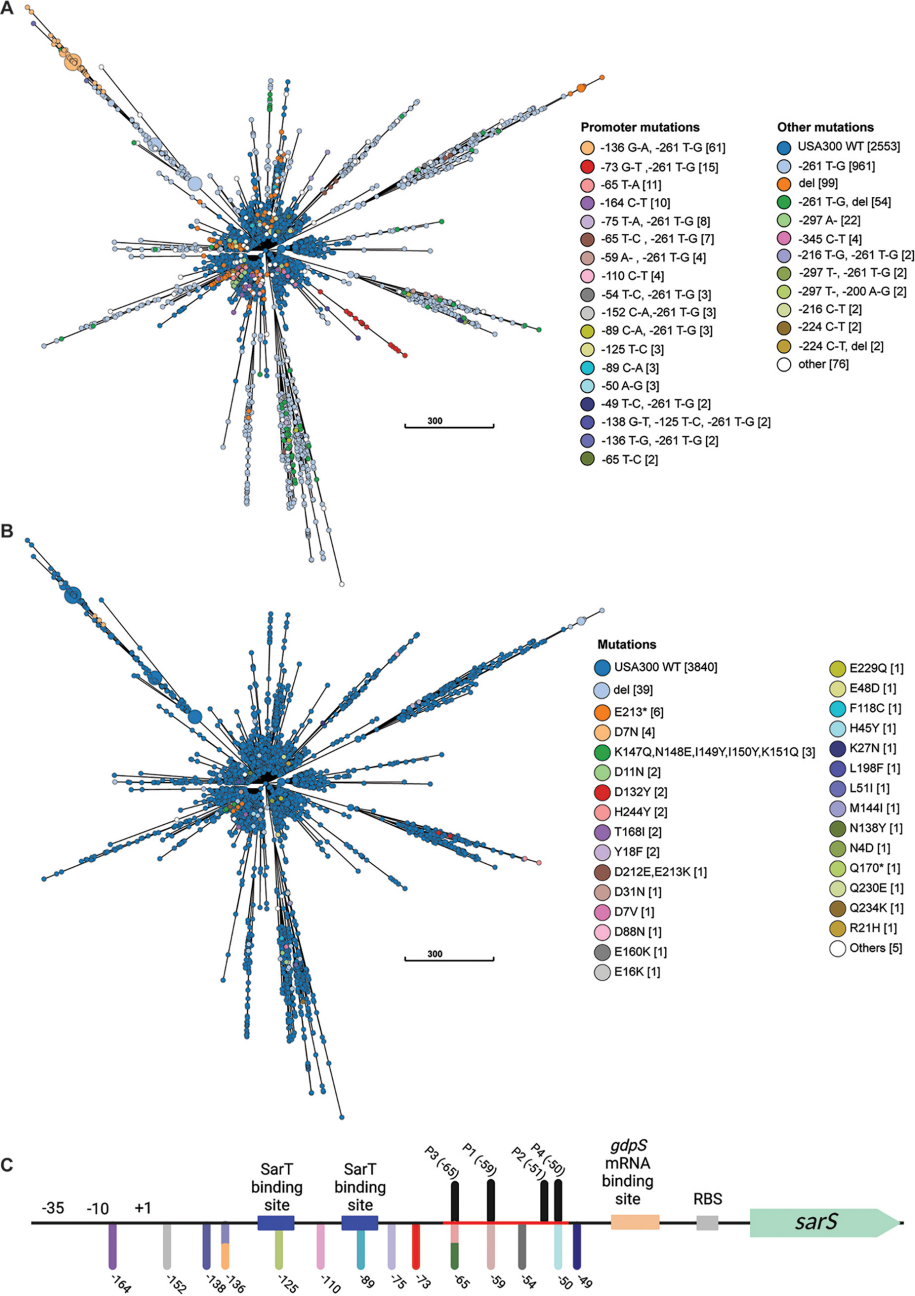
USA300 isolates harbor mutations upstream of *sarS*. Shown is a GrapeTree visualization of a cgMLST tree of USA300 genomes deposited in GenBank as of April 2022. Branch lengths correspond to the number of cgMLST locus differences. Genomes with ≤10 differences are collapsed into a single node. Nodes are sized proportionally to the number of genomes they represent. Each node is colored according to which mutation or combinations of mutations in the *sarS* promoter (A) or nonsynonymous mutations in the *sarS* coding region (B) were present. Promoter mutations indicate mutations downstream of the −35 RNA polymerase binding site, and other mutations are those found in the upstream intergenic region. (C) Schematic of *sarS* and upstream region with previously described features (37) and mutations found in the promoter region. Positions are numbered relative to the start of the coding sequence.

Having established that natural mutations in the *sarS* promoter can impact USA300 virulence, we next investigated if mutations in the *sarS* promoter/5′ UTR were widespread. We examined the *sarS* intergenic region and coding sequence across the USA300 genomes in GenBank for the presence of mutations (Fig. 6A). Mutations were broadly observed in the intergenic region (~35% of isolates), and mutations downstream of the −35 RNA polymerase binding site were also identified (~4% of isolates). These analyses also revealed that mutations in the *sarS* intergenic region are more pervasive than nonsynonymous mutations in the *sarS* gene (~35% versus ~2%) (Fig. 6B). Interestingly, we identified mutations in the 16-bp region of *sarS* DNA that harbors the P1, P2, P3, and P4 mutations (Fig. 6C). Additionally, there are mutations at the exact site of the P1, P3, and P4 mutations, further supporting the idea that mutations that alter *sarS* expression are being selected for in broader USA300 populations. These data indicate that mutations in the *sarS* promoter are not unique to our data set but widespread. The recurrence of these mutations across an extensive population suggests that expression of *sarS* could be a target of evolution in USA300.

## DISCUSSION

The bicomponent pore-forming leukocidins are a critical component of the S. aureus virulon (6, 48, 49), but a complete understanding of the networks that regulate their expression is lacking. In this work, we describe a screen that combined transcriptional reporters with transposon mutants to identify novel regulators of leukocidins. Among the mutants identified, we characterized and established SarS as a key repressor of *lukED* and *lukSF-PV* expression (Fig. 1 and 2). We demonstrated the impact of SarS in *ex vivo* and *in vivo* infection models and identified activation of Rot and direct binding to toxin promoters as two mechanisms by which SarS regulates toxins (Fig. 3 and 4). Additionally, we found that mutations in the *sarS* promoter occur naturally in clinical USA300 isolates (Fig. 5), suggesting selective pressure to alter *sarS* expression during infection.

In terms of toxin regulation, SarS was initially identified as a repressor of *hla*, an effect that was dependent on the absence of *sarA* (34). Throughout this study we did not see a profound upregulation of *hla* in the absence of *sarS*, which could be because *sarA* was present in all strains used. While this initial study helped us understand the role of SarS in virulence regulation and shed light on how the Agr system and SarA mediate regulation of virulence factors (34), we now show that SarS is also a regulator of *lukED* and *lukSF-PV*.

It has been shown that the leukocidin promoters are differentially regulated *in vitro* (32), with the *lukSF-PV* and *lukAB* promoters being the most active during postexponential phase. This differential promoter activation could be in part due to differential regulation by repressors and activators such as SarS. Interestingly, we observed differential regulation of the leukocidins by SarS, in which *lukED* and *lukSF-PV* are highly repressed while the other bicomponent pore-forming leukocidins are not significantly regulated by SarS. It stands to reason that a pathogen that is able to successfully infect numerous environmental niches within the human body must be able to fine-tune its virulence factors to adapt to whatever tissue it is inhabiting (28). The ability of S. aureus to differentially regulate the expression of the leukocidins would therefore be beneficial to the adaptability of the pathogen.

We found that SarS can repress the leukocidins both indirectly through the activation of Rot and directly through the binding of the toxin promoters. It holds true to a trend seen in S. aureus virulence regulation, where both direct and indirect regulation of target genes is often observed. Such is the case with Rot, which can both directly and indirectly repress leukocidins through the binding of promoters and the repression of the SaeRS system (15, 16, 18). The observation that Rot and SarS are closely intertwined in their regulation is interesting, as Rot has been shown to be an activator of SarS (38). However, Hsieh et al. found *rot* to be upregulated in the absence of *sarS*, suggesting that SarS is a repressor of Rot (50). The experiments conducted by Hsieh et al. were performed in a different background, a derivative of S. aureus strain NCTC 8325 that was cured of all prophages (50, 51), whereas the experiments conducted in our study were performed in a derivative of strain LAC, a representative of the CA-MRSA USA300 lineage. The many differences between these strains could account for the contrasting phenotypes observed with regard to *rot* regulation by SarS.

An interesting finding highlighted in this work is that USA300 clinical isolates harbor mutations in the *sarS* promoter that may lead to altered expression of *sarS* and decreased toxin expression and cytotoxicity. This finding sheds light on the importance of studying noncoding mutations to further understand S. aureus pathogenesis. These strains have likely evolved to thrive in patients, following an interesting trend seen in hospital-associated bloodstream infection: regulators of virulence factors are mutated to result in what may be less virulent strains *in vitro*. For example, the *agr* locus in hospital-associated MRSA isolates often have mutations that lead to attenuated *agr* activity (52–54). These observations highlight the need to understand how bacteria adapt under selective pressure in humans. The work presented herein demonstrates that one way S. aureus may adapt to infection is by altering cytotoxicity through the acquisition of mutations that impact *sarS* expression. There may be a give and take, a sacrifice of one type of virulence for the prioritization of other, that S. aureus must balance in order to become a successful pathogen. Alternatively, the bacterium may be adapting to better infect a certain type of host or to become more transmissible or have enhanced colonization. Hospitalized individuals often have impaired immune systems, and therefore, it may be advantageous for a nosocomial pathogen to downregulate certain virulence factors as to not overwhelm its host. Additionally, upregulation of virulence factors that aid in the adherence to medical devices can be beneficial in the hospital setting as well. While one role of SarS is to repress toxins, another key role is to activate the expression of *spa*. Protein A is a key virulence factor that promotes immune suppression and protects S. aureus from opsonophagocytic killing (55, 56). Therefore, S. aureus may upregulate *spa* expression *in vivo* through *sarS* promoter mutations at the expense of toxin production.

Altogether, the findings presented herein highlight the complex nature of toxin regulation in S. aureus. Additional studies are necessary to better understand the regulators, location, and host-derived signals at play during infection. We posit that such information, together with studies that incorporate contemporary clinical isolates that naturally differ in toxin regulation, are key to enable us to assemble a more complete picture of the regulation of toxins during infection and perhaps highlight potential opportunities for intervention.

## MATERIALS AND METHODS

### Ethics statement.

Buffy coats were obtained from anonymous blood donors with informed consent from the New York Blood Center. All animal experiments were reviewed and approved by the Institutional Animal Care and Use Committee of New York University Langone Medical Center (NYULMC). All experiments were performed according to NIH guidelines, the Animal Welfare Act, and U.S. federal law.

### Bacterial growth conditions.

S. aureus strains (Table 1) were routinely grown at 37°C on tryptic soy agar (TSA) or in tryptic soy broth (TSB). Escherichia coli bacteria were grown in Luria-Bertani broth. Agar and broth were supplemented with antibiotics as needed to the following concentrations: erythromycin to 5 μg/mL, chloramphenicol to 10 μg/mL (Cm10), and tetracycline to 4 μg/mL. Liquid cultures were grown in 5 mL of growth medium in 15-mL conical tubes and incubated at a 45° angle with shaking at 180 rpm, unless otherwise specified. For all experiments involving the growth of S. aureus, a 1:100 dilution of overnight cultures was subcultured into fresh medium.

**TABLE 1:** Bacterial strains used in this study

VJT #^*a*^	Strain name	Description	Reference
15.77	AH-LAC	Erm^s^ USA300 parent strain	44
31.81	JE2	Erm^s^ USA300 parent strain	30
79.82	JE2 pHC123 PlukSF_luc	JE2 carrying the pHC123 plasmid with the *lukSF* promoter driving the expression of *luc*	This study
67.43	AH-LAC *sarS*::*bursa*	AH-LAC carrying the *bursa aurealis* transposon in the *sarS* gene	This study
9.91	ALC1927(*sarS*::*erm*)	RN6390 containing erythromycin insertion into *sarS*	35
84.97	AH-LAC Δ*sarS*::*erm*	AH-LAC containing erythromycin insertion into *sarS*	This study
85.52	AH-LAC Δ*sarS*::*sarS*	AH-LAC Δ*sarS* complemented with *sarS* WT allele into original spot on the chromosome	This study
85.17	AH-LAC Δ*sarS* SaPI1::*sarS* WT	AH-LAC Δ*sarS* complemented with *sarS* WT allele into the SaPI1 site	This study
85.19	AH-LAC Δ*sarS* SaPI1::*sarS* P1	AH-LAC Δ*sarS* complemented with *sarS* ER00594 allele into the SaPI1 site	This study
25.77	AH LAC P*sae* GFP	AH-LAC carrying pOS1 plasmid with saeP1 promoter driving expression of superfolder *gfp*	19
27.61	AH LAC P*rot* GFP	AH-LAC carrying pOS1 plasmid with *rot* promoter and the *sod* RBS driving expression of superfolder *gfp*	19
32.98	AH LAC P*rot* 35 aa GFP	AH-LAC carrying pOS1 plasmid with the *rot* promoter driving expression of the first 35 amino acids of Rot fused to s*gfp*	32
34.46	AH LAC P*rnaIII* YFP	AH-LAC with pUC18-based plasmid with the *rnaIII* promoter driving expression of *yfp*	65
85.07	AH LAC Δ*sarS* P*sae* GFP	AH-LAC Δ*sarS* carrying pOS1 plasmid with saeP1 promoter driving expression of superfolder *gfp*	This study
85.08	AH LAC Δ*sarS* P*rot* GFP	AH-LAC Δ*sarS* carrying pOS1 plasmid with *rot* promoter and the *sod* RBS driving expression of superfolder *gfp*	This study
85.09	AH LAC Δ*sarS* P*rot* 35 aa GFP	AH-LAC Δ*sarS* carrying pOS1 plasmid with the *rot* promoter driving expression of the first 35 amino acids of Rot fused to s*gfp*	This study
85.10	AH LAC Δ*sarS* P*rnaIII* YFP	AH-LAC Δ*sarS* with pUC18-based plasmid with the *rnaIII* promoter driving expression of *yfp*	This study
67.64	AH-LAC + pOS1-pSarS-WT-sGFP	AH-LAC carrying pOS1 with *sgfp* under control of AH-LAC *sarS* promoter	This study
67.65	AH-LAC + pOS1-pSarS-P1-sGFP	AH-LAC carrying pOS1 with *sgfp* under control of ER00594 *sarS* promoter	This study
67.66	AH-LAC + pOS1-pSarS-P2-sGFP	AH-LAC carrying pOS1 with *sgfp* under control of ER02658 *sarS* promoter	This study
67.67	AH-LAC + pOS1-pSarS-P3-sGFP	AH-LAC carrying pOS1 with *sgfp* under control of ER04127 *sarS* promoter	This study
67.68	AH-LAC + pOS1-pSarS-P4-sGFP	AH-LAC carrying pOS1 with *sgfp* under control of ER05167 *sarS* promoter	This study
67.70	AH-LAC + pOS1-pSarS-RBS-sGFP	AH-LAC carrying pOS1 with *sgfp* under control of *sarS* RBS	This study
52.04	ER00594.3B	USA300 bloodstream clinical isolate containing the P1 mutation	46
52.30	ER02658.3B	USA300 bloodstream clinical isolate containing the P2 mutation	46
58.32	ER04127.3A	USA300 bloodstream clinical isolate containing the P3 mutation	46
52.54	ER05167	USA300 bloodstream clinical isolate containing the P4 mutation	46

a VJT # is the number given to the strain in our lab database. Inclusion of this number makes it easier to find strains should other lab reach out and request them.

### Construction of bacterial strains and plasmids.

For all the strains and oligonucleotides used in this study, see Tables 1 and 2.

**TABLE 2:** Oligonucleotides used in this study

VJT #	Name	Sequence
2439	rpoB_F	5′-GAACATGCAACGTCAAGCAG
2440	rpoB_R	5′-AATAGCCGCACCAGAATCAC
2740	rpoB_probe	5′-TACAGGTATGGAACACGTTGCAGCA
2734	lukE_F	5′-GGACTGACGACTAAAGATCCAAA
2735	lukE_R	5′-AATGAGCCATTGCCACCTAT
2736	lukE_probe	5′-TGGAGGTAATTTCCAGTCAGCACCA
2453	lukA_F	5′-GCTCAGGTGGTAAATTCGATTC
2454	lukA_R	5′-ACCGCTGGCAATTGTGTC
2702	lukA_probe	5′-TGGACGAACTTCATCAAATAGCTACTCCA
2435	lukS_F	5′-GCTGCAACATTGTCGTTAGG
2436	lukS_R	5′-GCGCCATCACCAATATTCTC
2737	lukS_probe	5′-TCACTCCTATTGCTACTTCGTTTCATG
2431	hlgA_F	5′-GGCAGTGGCTCATTCAACTAC
2432	hlgA_R	5′-CTTGACCATTCGGTGTAACG
2738	hlgA_probe	5′-CTGAAGTAGAAAGTCAGAACTCTAAAGGTG
2433	hlgCB_F	5′-GAGCTTACTTGCCCCTCTTG
2434	hlgCB_R	5′-TCGCTTCCTTTACCGATGTC
2739	hlgCB_probe	5′-TGCTAAAGCTGCTAACGATACTGAA
3015	hla_F	5′-AGATTCTTGGAACCCGGTATATG
3016	hla_R	5′-CTGTAGCGAAGTCTGGTGAAA
3011	hla_probe	5′-TGGCTCTATGAAAGCAGCAGATAACTTCC
3404	sarS_F	5′-CAATCCACCATAAATACCCTCAAAC
3405	sarS_R	5′-GCTGCGCGTCATCCATA
3406	sarS_probe	5′-AGAACGCTCAACTGAAGATGAAAGA
3214	PlukE_F	5′-CTTATTTGAAAAAAGCAAAAAAGATAGG
3215	PlukE_R_B	5′-CTTAAACATAAGTTTCACTTTCTTTC
3366	Phla_F	5′-GGCAAAATTTATTCCCGACG
3367	Phla_R_B	5′-CGTGTTTTCATTTTCATCATCC
3219	PlukS_F	5′-GATGGATTACTTATATTGCTAATAG
3220	PlukS_R_B	5′-GACCATAAAAATCATTTCCTTTC
3383	PlukS_R	5′-GACCATAAAAATCATTTCCTTTC
2306	PpurA_F	5′-AAAAGTTTTTCCGTACAATA
2307	PpurA_R_B	5′-ACATGTGAGCACCTCCAAGT
2305	PpurA_R	5′-ACATGTGAGCACCTCCAAGT
3216	PlukAB_F	5′-CATAATTTAAAATGCAGAAAGTAGTGTG
3217	PlukAB_R_B	5′-CATTGTTATAACCTTCTTTCGTATG
3220	PhlgA_F	5′-CGCTATTTGTCAGCCC
3221	PhlgA_R_B	5′-CACTTTCTTTCTATTTAATTTTAAGTTC
3407	Up_sarS_pIMAY_F	5′-CTCTAGAACTAGTGGATCCCCCGGGCCCTGTAGCGATTGGCTTAGGCTTAC
3408	Down_sarS_pIMAY_R	5′-GCTGGGTACCGGGCCCCCCCTCGAGCAGCTTTTGGAGCTTGAGAGTCATTAAG
3372	Up_sarS_pJC1111_F	5′-TGCATGCCTGCAGGTCGACTCTAGACGATATTATTAAAACAAAATGACCTCAC
3373	Down_sarS_pJC1111_R	5′-CGCGCCTGAATTCGAGCTCGGTACCTTATTCAAAAACAAGATGTAAATGATC
2314	pOS1_1_F	5′-TCAGGGGATAACGCAGGAAAG
2315	pOS1_1_R	5′-ATGAGCAAAGGAGAAGAACTTTTCA
2316	pOS1_2_F	5′-TGTTCTTTCCTGCGTTATCCCCTGATTCTGTGGATAACCGTATTACCGC
2317	pOS1_2_R	5′-CTGCAGCCAAGCTAGCTTGT
2318	pSarS_pOS1_F	5′-TGAAAAGTTCTTCTCCTTTGCTCATTGTTTTATCTCCTTGTATATGCACTTTATT
2319	pSarS_pOS1_R	5′-TGATTACAAGCTAGCTTGGCTGCAGCGATATTATTAAAACAAAATGACCTCACAA
N/A	sarS_RBS_pOS1_F	5′-TGAAAAGTTCTTCTCCTTTGCTCATTGTTTTATCTCCTTGCTGCAGCCAAGCTA GCTTGTAATCA
N/A	sarS_RBS_pOS1_R	5′-TGATTACAAGCTAGCTTGGCTGCAGCAAGGAGATAAAACAATGAGCAAAGGA GAAGAACTTTTCA
3006	pHC123_lukSF_F	5′-CCCCGTCGACAGATGGATTACTTATATTGCTAATAG
3007	pHC123_lukSF_R	5′-CCCCGGTACCCTTTATAAATTTTATTACATTTTTATATTAAACC

**(i) Reporter strains.** *(a) AH-LAC P*lukSF_*luc* *and regulatory library plus PlukSF_luc.* The backbone of the P*lukSF_luc* plasmid originated from the plasmid pHC123 (33) and was cut at the SalI and KpnI cut sites before being ligated with the *lukSF* intergenic region and being transformed into DH5α and electroporated into AH-LAC. The primers pHC123_*lukSF*_F and pHC123_*lukSF*_R were used. The promoter reporter library was generated by phage transduction using phage 80α lysate from the AH-LAC P*lukSF_luc* strain.

The regulatory library was grown overnight in 400 μL of TSB in a round-bottom deep-well plate. In the morning, 390 μL of fresh TSB was inoculated with 10 μL of the overnight culture and grown at 120 rpm until an optical density at 600 nm (OD_600_) of 1 was reached. Next, 5 μL of 1 M CaCl_2_ and 100 μL of phage lysate were added to each well, and the plate was left at room temperature for 20 min. We added 40 μL of 1 M sodium citrate, and 10 μL was spot platted onto TSA plus Cm10 and grown overnight. Colonies were picked from this plate and grown overnight, and then 50 μL of the overnight culture was added to 50 μL of 20% glycerol and frozen down for further use.

*(b) pSarS-WT-sGFP, pSarS-P1-sGFP, pSarS-P2-sGFP, pSarS-P3sGFP, pSarS-P4-sGFP, and pSarS-RBS-sGFP.* To make *sarS* promoter reporter strains, the vector containing *sgfp* (superfolding GFP) was amplified from pOS1sGFP-P*sarA*-*sod*RBS (19). The vector was digested with SmaI (inside the SarS promoter sequence) and amplified in two pieces for Gibson assembly so that just the backbone and *sgfp* were present (no promoter or RBS). This was done using primers pOS1_1_F and pOS1_1_R for one piece and pOS1_2_F and pOS1_2_R for the other. For inserts PWT and P1 to P4, the entire intergenic region between *sirC* and *sarS*, with homology to pOS1, was amplified using primers pSarS_pOS1_F and pSarS_pOS1_R. Genomic DNA for amplification was used as follows: PWT, AH-LAC; P1, ER00594; P2, ER02658; P3, ER04127; and P4, ER05167. For RBS control, the oligonucleotides sarS_RBS_pOS1_F and sarS_RBS_pOS1_R were resuspended at 100 μM in annealing buffer (10 mM Tris [pH 7.5 to 8.0], 50 mM NaCl, 1 mM EDTA) and placed in a 94°C heat block for 2 min. The heat block was then turned off and cooled gradually (45 to 60 min), allowing for the oligonucleotides to anneal. For Gibson assembly, 2× Gibson master mix (Fisher Scientific), the genomic insert, and the vector backbone were incubated at 50°C for 1 h. Following Gibson assembly, plasmids were heat transformed into E. coli IM08B and electroporated into AH-LAC.

*(c) Psae-GFP, Prot-GFP, Prot35-GFP, and PrnaIII-GFP.* Construction of the regulator promoter reporter strains was as previously described (32). The plasmids containing the GFP reporter fusions were electroporated into AH-LAC Δ*sarS* and selected for with 10 μg/mL of chloramphenicol.

**(ii) Disruption/deletion strains.** AH-LAC *sarS*::*bursa* was generated by phage transduction using phage 80α lysate from the JE2 Nebraska Transposon Mutant Library NE165 strain. The AH-LAC Δ*sarS*::*erm* strain was constructed by phage transduction using phage 80α lysate from the strain ALC1927 (35).

**(iii) Complementation strains.** AH-LAC Δ*sarS*::*sarS* was made by cloning the *sarS* allele into the pIMAY* plasmid (45) and integrating it into the original site in the chromosome. The following primers were used to amplify upstream and downstream of *sarS* with 25 bp of homology to pIMAY* for Gibson cloning: Up_*sarS*_pIMAY_F and Down_*sarS*_pIMAY_R. For Gibson assembly, 2× Gibson master mix (Fisher Scientific), the genomic insert, and the vector backbone were incubated at 50°C for 1 h. The plasmid was transformed into IMO8B and then electroporated into AH-LAC.

Δ*sarS* SaPI1::*sarS* wild type and Δ*sarS* SaPI1::*sarS* P1 were created by complementing the AH-LAC Δ*sarS*::*sarS* strain using the pJC1111 plasmid (47), which integrates a single copy of the complementing gene into the SaPI1 site. *sarS*, along with the upstream and downstream intergenic regions, was amplified from AH-LAC (wild-type allele) or the clinical isolate containing the P1 mutation (ER00594). The following primers were used to amplify upstream and downstream of *sarS*, with cut sites at KpnI and XbaI, and 25 bp homology to pJC1111 for cloning with Gibson: Up_*sarS*_pJC1111_F and Down_*sarS*_pJC1111_R. For Gibson assembly, 2× Gibson master mix (Fisher Scientific), the genomic insert, and the vector backbone were incubated at 50°C for 1 h. Following Gibson assembly, the plasmids were transformed into DH5α and electroporated into AH-LAC Δ*sarS*.

### Transcriptional regulator screen.

The library was grown in a 1-mL volume in round-bottom deep-well block plates (Axygen; P-DW-20-C) in TSB plus 10 μg/mL of chloramphenicol. The following morning, the bacteria were subcultured 1:100 in a 1-mL volume in round-bottom deep-well block plates in TSB plus 5 μg/mL of chloramphenicol. The plates were incubated at 37°C with shaking at 180 rpm for 5 h. Next, 100 μL was removed and added to a clear-bottom round plate to measure OD and a white-bottom flat plate (Corning) to measure luminescence, which was assessed with a PerkinElmer EnVision 2130 multilabel reader. A total of 0.15 mg of luciferin was added to each well of the plate in a 10-μL volume, the plate was incubated in the dark for 30 min, and then luminescence was measured. All luminescence values were OD normalized.

### RNA isolation.

Bacteria were subcultured for 3 and 5 h in 5 mL of TSB before being spun down and resuspended in 1 mL of RNA Stat-60 (Amsbio). Samples were bead beated in lysing matrix B tubes (MP Bio) using the FastPrep and spun down for 10 min at 12,000 × *g*. The upper layer was collected and 200 μL of chloroform was added. The samples were incubated at room temperature for 3 min before being spun down for 15 min at 12,000 × *g*. The aqueous phase was removed, and 0.5 mL of isopropanol was added to precipitate RNA. RNA was washed twice with 70% ethanol, air dried, and resuspended in RNase-free water. RNA (10,000 ng) was DNase treated (TURBO DNA-fee kit; Invitrogen Ambion).

### qRT-PCR.

DNase-treated samples were converted into cDNA with the SuperScript III first-strand synthesis kit (Thermo Scientific). Next, 1 μL of cDNA was added to TaqMan probes and universal PCR master mix. Transcript levels were measured using the QuantStudio 3 system. Genes were normalized to the *rpoB* housekeeping gene and reported as threshold cycle (2^−Δ^*^CT^*). Probes used were PrimerTime 5′ Hex and 3′ BHQ-1.

### Exoprotein isolation, Coomassie staining, and immunoblotting.

The proteins in the culture supernatants of bacteria grown for either 3 or 5 h were precipitated and analyzed as previously described (16). Briefly, following OD normalization, the cultures were spun down and 1.4 mL of supernatant was added to 140 μL of trichloroacetic acid (TCA) and left overnight at 4°C. The precipitated proteins were sedimented, washed, dried, resuspended in 8 M urea, and left at room temperature for 30 min. Next, 2× SDS loading buffer was added and the mixture was boiled for 10 min. Proteins were separated on a 12% SDS-PAGE gel, transferred to nitrocellulose membranes, and probed with indicated primary antibodies.

For the Rot and SaeR Western blots, 5-mL cultures were grown and spun down and the pellet was resuspended in lysis buffer (50 mM Tris, 10 mM MgCl_2_, 1 mM CaCl_2_) with 5% lysostaphin, 1% HALT protease inhibitor, 1% RNase A, and 4% DNase and incubated at 37°C for 30 min. The samples were boiled for 10 min and then spun down at 15,000 × *g* for 20 min. The top layer was filtered and 4× SDS sample buffer was added before boiling the samples again.

Immunoblotting was performed with monoclonal antibodies against LukE (1:5,000) and LukF-PV (1:5,000), which were detected with a fluorescent Alexa Fluor 680-conjugated anti-mouse antibody (1:25,000; Invitrogen). Alpha-toxin (1:5,000), Rot (1:2,000), and SaeR (1:2,000) were detected with a polyclonal antibody and a fluorescent Alexa Fluor 680-conjugated anti-rabbit antibody (1:25,000; Invitrogen).

### GFP regulator reporter assay.

GFP assay with the regulator promoters was performed as previously outlined by Balasubramanian et al. (32). Briefly, strains containing reporter plasmids were grown overnight in TSB with 10 μg/mL of chloramphenicol in round-bottom 96-well plates. The following morning, cultures were subcultured in 96-well black flat-bottom plates (Corning) and grown for the indicated time. Fluorescence was measured with a PerkinElmer EnVision 2130 multilabel reader.

### SarS purification.

Full-length *sarS* was amplified from AH-LAC, adding XhoI and BamHI cut sites, and cloned into pET15b. The plasmid was transformed into BL21-DE3g. The expression strain was grown in 400 mL of LB broth with 100 μg/mL of ampicillin at 37°C and 250 rpm until the cultures reached an OD_600_ of 0.6. The culture was induced with 1 mM isopropyl-β-d-thiogalactopyranoside (IPTG) and grown for 4 h at 37°C and 250 rpm. The culture was spun down and resuspended in 20 mM Tris (pH 7.5), 300 mM NaCl, and 10% glycerol. Cells were lysed in 1× protease inhibitor (Pierce protease inhibitor cocktail) and sonicated on ice. Bugbuster (Millipore) at 1× was added, and the cultures were incubated at room temperature for 35 min. Following incubation on ice for 15 min and centrifugation, the supernatant was filtered through a 0.2-μm filter. The protein was purified using a HisTrap HP column on an AKTA system, eluting with a linear gradient in elution buffer (20 mM Na_2_HPO_4_, 500 mM NaCl, 400 mM imidazole [pH 7.4]). Purified protein was dialyzed in 10% glycerol in TSB.

### Promoter pulldown assays.

The promoter pulldown assay protocol was adapted from that of Sause et al. (57). We generated PCR products for each promoter using oligonucleotides containing biotinylated labels and purified the PCR products (Qiagen). M-280 streptavidin Dynabeads (Invitrogen; 11205D) were washed and resuspended in wash buffer (2 M NaCl, 1 mM EDTA, 10 mM Tris [pH 7.5]). The beads were incubated with 800 fmol of each DNA fragment for 30 min at room temperature on a rotisserie. The samples were washed three times with wash buffer before being resuspended in binding buffer (25 mM Tris-HCl, 0.1 mM EDTA, 75 mM NaCl, 10% glycerol, 1 mM dithiothreitol [DTT; pH 7.5]). SarS at a concentration of 100 nM and 5 μg of poly(dG·dC) were added to the beads and mixed on a shaking platform for 15 min at 30°C and 550 rpm. For competition experiments, 800 fmol of nonbiotinylated DNA was mixed with the DNA-conjugated beads before the addition of SarS. Following incubation, beads were washed twice with binding buffer, resuspended in 1× SDS sample buffer, and boiled for 10 min. Following electrophoresis, gels were transferred to a nitrocellulose membrane and probed with an anti-His antibody (1:2,000; Cell Sciences; CSI20563B) and detected with a fluorescent Alexa Fluor 680-conjugated anti-mouse antibody (1:25,000; Invitrogen).

### Cytotoxicity assay and extracellular hPMN infections.

Human polymorphonuclear leukocytes (hPMNs) were isolated using a Ficoll-Paque method and cytotoxicity assays were performed as described by Reyes-Robles et al. (58). Briefly, PMNs were seeded at 2 × 10^5^ cells per well in RPMI medium without phenol red (Fisher Scientific) supplemented with 10% heat-inactivated fetal bovine serum (FBS; Gemini Bio-Products). Either 1.25% or 2.5% supernatant was added (percentage is the percentage of total reaction volume that was culture supernatant). To measure cell viability, the metabolic dye CellTiter (Promega) was added at a final concentration of 10% per well and incubated for 2 h at 37°C and 5% CO_2_. Absorbance at 492 nm was measured using the PerkinElmer EnVision plate reader. For extracellular infections, bacteria were subcultured for 3 h in 5 mL of TSB, washed twice with PBS, and normalized to an OD_600_ of 1. hPMNs were added to a flat-bottom tissue culture treated plate at 2 × 10^5^ and incubated at room temperature for 30 min. Bacteria were added at a multiplicity of infection (MOI) of 100. After a 2-h infection at 37°C and 5% CO_2_, hPMN viability was determined by lactate dehydrogenase (LDH) release (CytoTox-ONE homogenous membrane integrity assay; Promega), measured using the PerkinElmer EnVision plate reader.

### Animal housing conditions.

Animals received PicoLab rodent diet 20 (LabDiet) and acidified water and were housed under normal lighting cycle conditions (12 h on/12 h off) and a temperature of 70°F.

### Murine intraperitoneal infection.

Bacteria were subcultured for 3 h in 5 mL of TSB, washed twice in PBS, and OD normalized. Eight-week-old C57BL/6J female mice (Jackson Laboratory) were injected with 200 μL of bacteria intraperitoneally. Mice were monitored multiple times a day for 48 h and euthanized upon severe signs of mortality or excessive weight loss.

### Analysis of USA300 genomes in GenBank.

A total of 45,270 S. aureus genomes from human biosamples were downloaded in April 2022 using NCBI data sets version 13.7.0. Genomes were deduplicated such that only the first genome per biosample was retained for further analysis. In addition, biosamples and genomes originating from the Mount Sinai Health System were removed, leaving 26,792 genomes after filtering. The multilocus sequence type (MLST) of each genome was determined using *mlst* (https://github.com/tseemann/mlst) and PubMLST (59) and used to assign the USA group of each isolate as defined by David et al. (60). To obtain a comprehensive list of mutations seen in *sarS* and the *sarS* promoter, the sequence of USA300 FPR3757 (GenBank accession number NC_007793) was used as the wild type and aligned using BLASTn. Subsequently, each of the 26,792 GenBank genomes was compared (61). For all the USA300 GenBank isolates, core gene MLST (cgMLST) schemes for each were determined using chewBBACA (version 2.8.5) (62) and the cgMLST scheme available at https://cgmlst.org/ncs (63). cgMLST trees were visualized using GrapeTree (64).

### sGFP reporter assay.

Bacteria were subcultured from overnight cultures 1:100 in 5 mL of TSB plus 5 μg/mL of chloramphenicol. After 5 h of growth, cultures were spun down and resuspended in PBS. Samples were serially diluted 1:2 × 6 (samples were serially diluted 1:2 six times) in 100 μL in a 96-well flat-bottom plate. GFP signal was determined using a PerkinElmer plate reader. Background signal was subtracted from PBS-only controls.

### Statistical methods.

Prism software (GraphPad, Inc.) was used to perform statistical analysis. Two-way analysis of variance (ANOVA) was utilized for qRT-PCR, promoter pulldown assays, GFP reporter assay, cytotoxicity, hPMN infection, luminescence screen hits, and sGFP assays. The statistical significance of difference between survival curves was determined by the log rank Mantel-Cox test.
